# Classifying flow cytometry data using Bayesian analysis helps to distinguish ALS patients from healthy controls

**DOI:** 10.3389/fimmu.2023.1198860

**Published:** 2023-08-01

**Authors:** Saskia Räuber, Christopher Nelke, Christina B. Schroeter, Sumanta Barman, Marc Pawlitzki, Jens Ingwersen, Katja Akgün, Rene Günther, Alejandra P. Garza, Michaela Marggraf, Ildiko Rita Dunay, Stefanie Schreiber, Stefan Vielhaber, Tjalf Ziemssen, Nico Melzer, Tobias Ruck, Sven G. Meuth, Michael Herty

**Affiliations:** ^1^ Department of Neurology, Medical Faculty, Heinrich Heine University of Düsseldorf, Düsseldorf, Germany; ^2^ Department of Neurology, Center of Clinical Neuroscience, University Hospital Carl Gustav Carus, Dresden University of Technology, Dresden, Germany; ^3^ Institute of Inflammation and Neurodegeneration, Otto-von-Guericke University Magdeburg, Magdeburg, Germany; ^4^ Department of Neurology, Otto von Guericke University, Magdeburg, Germany; ^5^ Department of Mathematics, Institute of Geometry and Applied Mathematics, RWTH Aachen University, Aachen, Germany

**Keywords:** ALS, Bayesian analysis, flow cytometry, immune system, mathematical modeling

## Abstract

**Introduction:**

Given its wide availability and cost-effectiveness, multidimensional flow cytometry (mFC) became a core method in the field of immunology allowing for the analysis of a broad range of individual cells providing insights into cell subset composition, cellular behavior, and cell-to-cell interactions. Formerly, the analysis of mFC data solely relied on manual gating strategies. With the advent of novel computational approaches, (semi-)automated gating strategies and analysis tools complemented manual approaches.

**Methods:**

Using Bayesian network analysis, we developed a mathematical model for the dependencies of different obtained mFC markers. The algorithm creates a Bayesian network that is a HC tree when including raw, ungated mFC data of a randomly selected healthy control cohort (HC). The HC tree is used to classify whether the observed marker distribution (either patients with amyotrophic lateral sclerosis (ALS) or HC) is predicted. The relative number of cells where the probability q is equal to zero is calculated reflecting the similarity in the marker distribution between a randomly chosen mFC file (ALS or HC) and the HC tree.

**Results:**

Including peripheral blood mFC data from 68 ALS and 35 HC, the algorithm could correctly identify 64/68 ALS cases. Tuning of parameters revealed that the combination of 7 markers, 200 bins, and 20 patients achieved the highest AUC on a significance level of p < 0.0001. The markers CD4 and CD38 showed the highest zero probability. We successfully validated our approach by including a second, independent ALS and HC cohort (55 ALS and 30 HC). In this case, all ALS were correctly identified and side scatter and CD20 yielded the highest zero probability. Finally, both datasets were analyzed by the commercially available algorithm ‘Citrus’, which indicated superior ability of Bayesian network analysis when including raw, ungated mFC data.

**Discussion:**

Bayesian network analysis might present a novel approach for classifying mFC data, which does not rely on reduction techniques, thus, allowing to retain information on the entire dataset. Future studies will have to assess the performance when discriminating clinically relevant differential diagnoses to evaluate the complementary diagnostic benefit of Bayesian network analysis to the clinical routine workup.

## 1 Introduction

Single-cell analysis is an emerging tool that enables the investigation of individual cells, providing insights into cellular heterogeneity, cellular behavior, and cell-to-cell interactions. With the advent of advanced technologies such as single-cell sequencing, proteomics, and high-resolution imaging, there has been a rapid expansion of single-cell analysis applications in diverse medical fields. However, the complexity and variability of single-cell data present significant challenges in terms of data analysis, interpretation, and integration.

Multidimensional flow cytometry (mFC) presents a well-established, widely available, and cost-effective method allowing for the broad characterization of different immune cell populations. Historically, analysis of mFC data largely relied on manual gating strategies. Biological knowledge provides the basis for the gating process which can be advantageous in certain cases, however, it also comes with several limitations, e.g., the limited possibility to discover novel cell populations and marker dependencies. In addition, manual gating is a time-consuming process prone to investigator bias. The development of (semi-)automated gating strategies and analysis tools tried to overcome these roadblocks with multiple algorithms achieving outcomes comparable, or even exceeding, manual strategies (1–3). In this context, different stages of the manual analysis pipeline were automated. For example, algorithms to identify and cluster cell subsets, to predict certain outcome measures, or algorithms for sample classification (diseased vs. non-diseased) were developed (1). These algorithms identify cell populations based on their similarity. Subsequently, the outcome of interest (e.g., diseased vs. non-diseased) is predicted based on information obtained from cellular subsets (3). A potential limitation to this approach is introduced by the need to reduce the dataset to specific cellular subsets, thereby potentially losing upstream biological information.

We here propose a novel unbiased approach to the analysis of mFC data using Bayesian analysis. This approach was designed as a classification task that allows to discriminate between two or more groups of data. To test this strategy, we investigated standard mFC data from diseased and non-diseased subjects. We chose to study immune cells from patients with amyotrophic lateral sclerosis (ALS) and healthy controls (HC). The latter were used to construct a data tree. Next, HC and ALS datasets were tested against this data tree. The utility of Bayesian analysis to distinguish ALS patients from HC when using peripheral blood (PB) mFC raw data as input for the algorithm was confirmed in two independent cohorts with different mFC panels. Taken together, Bayesian analysis might provide a strategy for automated interpretation of mFC data without the need for data dimensionality reduction.

## 2 Methods

### 2.1 Multidimensional flow cytometry data acquisition

**Figure 1:**
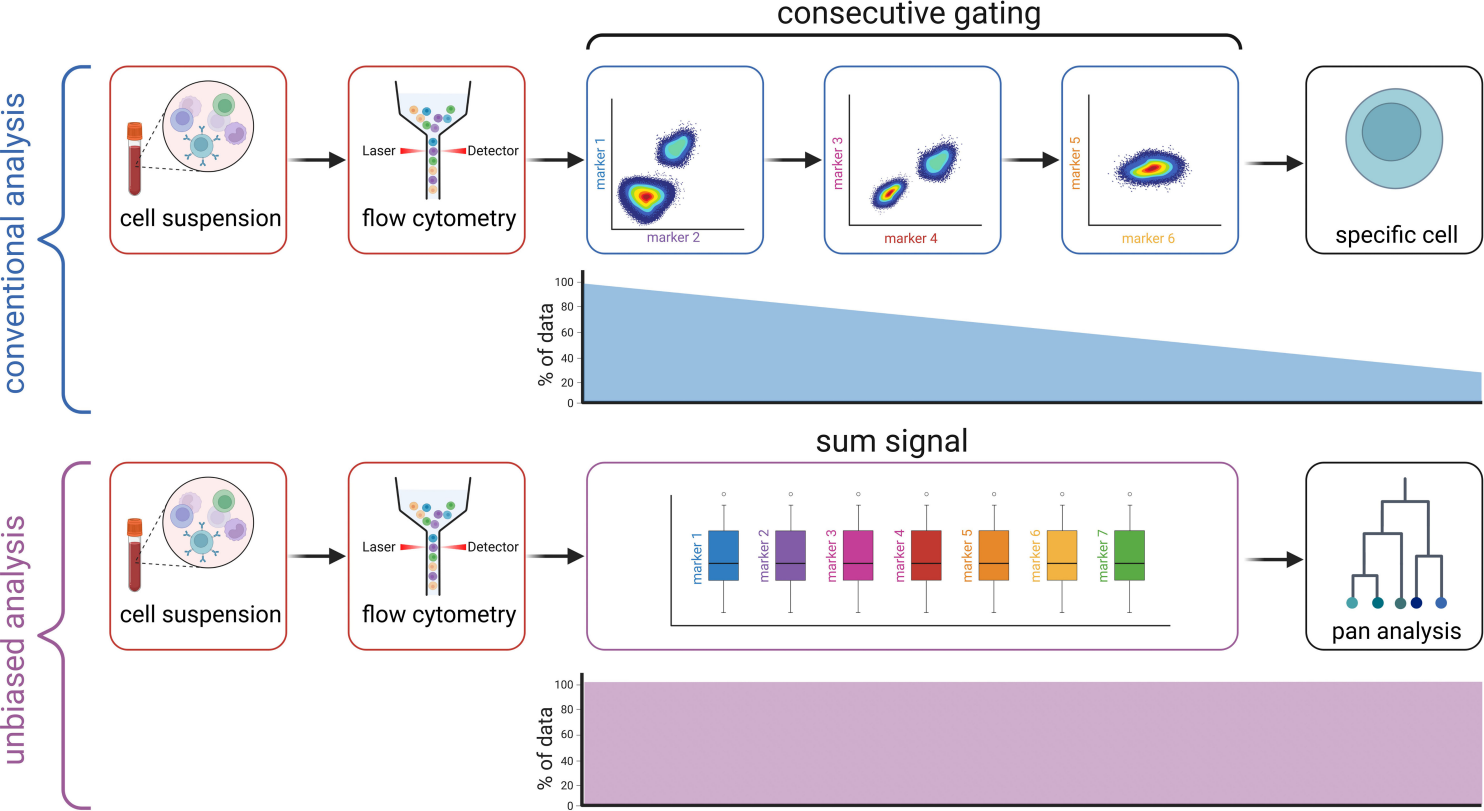
Workflow compared to conventional gating. Created with BioRender.com.

MFC raw data (Flow Cytometry Standard [FSC]) from a previously published study analyzing immune cell changes in the PB of ALS patients compared to HC were used (4). In total, cohort I (Dresden cohort) consisted of 68 ALS patients and 35 HC. In this cohort, the following markers were assessed for all patients: FSC-A, SSC-A, CD11b, CD45RA, CD45RO, CD25, CD38, CD3, CD20, CD8, CRTH2, CCR7, CD11c, CD4. Sample preparation and the flow cytometry staining protocol have been previously described in detail (4). In brief, PB samples were collected in lithium-heparin tubes (Sarstedt) and Peripheral Blood Mononuclear Cells (PBMCs) were isolated by Ficoll–Hypaque (Biochrom) density centrifugation. Surface staining with antibodies targeting the above-mentioned antigens (BD Bioscience) was performed. Samples were analyzed using a LSR-Fortessa (BD Biosciences). To validate our approach, we included a second cohort (Magdeburg cohort) containing 55 ALS patients and 30 HC. For this cohort, blood was collected in tubes containing Ethylenediaminetetraacetic acid (EDTA) (BD Vacutainer) and further processed as previously described (5). Briefly, blood was lysed with 1X red blood cell lysing buffer (BioLegend, 10X) and washed before surface immunostaining. The following markers were analyzed for all patients: FSC-A, SSC-A, CD16, HLA-DR, CX3CR1, CCR2, CD86, CD14, and CD66b, CD19, CD56, CD3 (serving as Dump channel). All FSC files were uploaded to the platform OMIQ from Dotmatics (www.omiq.ai, www.dotmatics.com) and were converted into csv files containing scaled values for every individual channel and each single cell. The csv files were then saved in the Excel standard format, which was used as input for the analysis using graphical models, more precisely, Bayesian networks. The workflow compared to a conventional mFC analysis is illustrated in **Figure 1**. The study was conducted in accordance with the Declaration of Helsinki and was approved by the local ethics committees (Ethikkommission an der Technischen Universität Dresden (EK393122012) and Ethik-Kommission der Otto-von-Guericke-Universität in Magdeburg (07/17 and 11/21)).

### 2.2 Mathematical modeling

#### 2.2.1 Summary

We propose a mathematical model for the dependencies of the different obtained markers using a graphical model, more precisely, a Bayesian network also called directed model or belief propagation network, see e.g (6–12). The purposes of constructing such a Bayesian network is twofold. On the one hand, we use the network for structured learning, i.e., for designing dependencies across the markers, and on the other hand, we use it for inference on new patient data, i.e., classifying whether or not the observed marker distribution is predicted by the Bayesian network.

#### 2.2.2 Methodology

We propose to analyze the data available through the mFC data acquisition in the following way: We assume that the two cohorts of patients, ALS and HC, can be differentiated by their marker distribution. For each cohort, we are given a set of data points X_{i,j} where i indexes the number of cells and j indexes the number of markers. The value of X_{i,j} denotes the measured number of markers j of cell i. The range of i is typically of the order of 10^6 to 10^7 while the range of j = 1,…,J for our mFC data. Since the number of cells is large compared to the number of markers, we assume that each cell i is a realization of an *unknown* J-dimensional random variable x, equivalently, each data set (X_{i,1}, … X_{i,J}) is considered as single realization of the random variable x on the probability space R^J and the canonical set of Borel measures. For simplicity, we assume that x is absolutely continuous with respect to the Lebesgue measure. We propose to develop a model for the probability density x → p(x) of the random variable x, i.e., R(x = **x**) = p(x). We assume that the cohorts ALS and HC can be distinguished by their respective (unknown) probability density function p.

##### 2.2.2.1 Modeling and approximation of p

The probability density function p: R^J → [0,1] is an unknown function in J variables. We use marginal statistics of x to derive (an approximation to) p. We approximate the j = 1,.J, marginal probabilities of x (over R) by


$$\begin{array}{c} \text{p}\_\text{j}\left(\text{y}\right)\;=\;\text{INTEGRAL}\;\text{p}\left(\text{x}\_1,\;\ldots \;\text{x}\_\left\{\text{j}-1\right\},\;\text{y},\;\text{x}\_\left\{\text{j}+1\right\},\;\ldots \;,\;\text{x}\_\left\{14\right\}\right)\\ \;\text{dx}\_1\;\ldots \;\text{dx}\_\left\{\text{j}-1\right\}\;\text{dx}\_\left\{\text{j}+1\right\}\;\ldots \;\text{dx}\_\left\{\text{J}\right\} \end{array}$$

using the data on markers j over all cells, see **Supplementary Figure 1**. Due to the large number of cells, we expect this approximation to be accurate. However, in order to obtain the model of the cohort we require to have **p**. This function is still a J-dimensional probability distribution that we assume can be approximated by products of first and second-order probability distributions Q(x_j|x_k), for some j, k, see formula (1) in Ref 7:


p(x) ∼ q(x)=PRODUCT_i Q(x_m(i) / x_m(j(i))

Mathematically, the obtained approximate probability distribution **x → q(x)** is a Bayesian network. Bayesian networks belong to the class of graphical models for probability distributions and assign each event a certain probability, see also (6) and references therein. Note that the previous assumption implies that each marker x_{m(i)} is assumed to be conditioned on at most one other marker x_m(j(i)). This induces a dependence tree between the markers. It also breaks the complexity of the problem, having now only to determine the corresponding marker relations. From a numerical point of view, a basic method for constructing such a Bayesian network is given by the Chow-Liu algorithm (7).

##### 2.2.2.2 Algorithm to obtain q

In the following, we outline the basic algorithmic steps to construct the approximation q. At first, we normalize the data X column-wise. Then, we construct the marginal distributions p_j for each marker j = 1,…, J. Those are then binned in order to obtain discrete distributions with N_x number of bins for each marker j. Theoretically, we could approximate the obtained marginal distributions by (binned) Gaussian distributions, but the results did not show any improvement using this procedure. Using the now discrete marginal distributions we apply the Chow-Liu algorithm and obtain the mutual information distribution of each combination of markers, see formula 2 in Ref 7. This requires to compute n(n-1)/2 distributions on the bins Nx, where n is the number of markers. Then, Kruskals algorithm is applied to obtain the minimal spanning tree where the weights are given by the mutual information. The tree then defines the indices m(i) and m(j(i)) in the probability density q. The probability Q is obtained from the mutual information of the previous step. This fully defines q. Assuming that marker distributions are different between the ALS and HC cohort, we repeat the previous approach for each cohort separately and obtain corresponding probability densities q_ALS and q_HC, respectively. This completes the structural learning part.

##### 2.2.2.3 Inference on new patient data

Including a new patient with cell-marker data Y_{i,j}, we propose the following method to classify this patient. We compute the likelihood that Y_{i,j} is a realization of x using the Bayesian network q. For each cell i, the (normalized) values Y_{i,1}, … Y_{i,J} represent a point in the J-dimensional marker space. Hence, the probabilities for i = 1,…, q(Y_{i,1}, … Y_{i,J}) is evaluated using the previously constructed probability density. If this probability is equal to zero, we assume that this cell data is an unlikely realization of x. Hence, we propose to solely count the relative number of cells for this patient that leads to a probability of zero and classify the patient as ALS or HC using this relative number as outlined in sections 3.2. and 3.3.

##### 2.2.2.4 Inference on relevant markers

Since q is composed of first and second-order probability density functions Q(x_j|x_k) a statistic on the markers that yield a zero probability is obtained. Here, we count the relative number of occurrences of cell markers j and k, respectively, that lead yields Q equal to zero (and hence leads to q equal to zero). For the new patients the statistics of those indices is reported in sections 3.2. and 3.3.

### 2.3 Citrus (cluster identification, characterization, and regression)

A commercially available algorithm to classify mFC data (CITRUS) was applied to the dataset to compare the performance of Bayesian analysis with a previously validated approach. Citrus presents a data-driven approach to identify stratifying cell subpopulations in a mFC dataset (2). To run Citrus, the OMIQ software from Dotmatics (www.omiq.ai, www.dotmatics.com) was used. For this, FCS files were uploaded to the OMIQ platform and the information on the group (diseased vs. non-diseased) was added to the file metadata. As the algorithm is not constructed for very high cell numbers, subsampling of cells (10 000 per sample) was performed prior to initiating the Citrus workflow. For Citrus, default settings were used and ‘medians’ was selected as ‘Feature Type’.

### 2.4 Visualization and statistical analysis

The software ‘GraphPad Prism’ (version 9.0.0) was used for downstream analyses and data visualization. The number of zeros was compared between the ALS and HC group. As normal distribution of data could not be assumed based on the D’Agostino & Pearson test, groups were compared using the Mann-Whitney U test. A p-value of ≤ 0.05 was considered significant. The performance of the classification based on sensitivity and specificity represented by the area under the curve (AUC) was assessed by receiver operating characteristic (ROC) analysis. Figures were created with the software ‘Inkscape’ (version 1.2) (13).

## 3 Results

### 3.1 Basic cohort characteristics

MFC data from two German centers (Dresden and Magdeburg) were used. In total, data of 123 ALS patients and 65 HC were included. Basic demographic and clinical characteristics are displayed in **Table 1**. The Dresden cohort was previously described in detail (4).

**Table 1:** Basic cohort characteristics.

	ALS (D)	HC (D)	ALS (M)	HC (M)
Number of individuals	68	35	55	30
Sex [% female]	58.8	38.7	44.0	46.7
Age at sample collection (median with range) [years]	66.9 (41.4-85.2)	61.7 (26.0-84.0)	64.5 (23.0-81.0)	64.5 (23.0-82.0)
Disease duration (median with range) [years]	1.7 (0.1-12.0)	n/a	1.67 (0-15.33)	n/a
ALSFRS-R (median with range)	36 (0-47)	n/a	37 (14-47)	n/a

ALS, Amyotrophic lateral sclerosis; ALSFRS-R, revised Amyotrophic Lateral Sclerosis Functional Rating Scale; D, Dresden; HC, healthy controls; M, Magdeburg; n/a, not applicable.

### 3.2 Unsupervised analysis of mFC data using Bayesian analysis can differentiate ALS patients from HC

Previous studies have highlighted the potential of mFC in the diagnostic workup of neurological disorders (3, 14, 15). Given the fact that conventional gating of mFC data is usually time consuming and investigator-dependent, different (semi-automated) approaches have been introduced to the analysis of mFC data. As those algorithms often focus on specific cell populations (and their relative differences) and the number of input cells is limited, we developed a mathematical model for the dependencies of the different obtained markers using a Bayesian network. The model uses raw mFC data of a randomly selected control cohort (HC, marker intensities of every single cell in the dataset) as input to create a Bayesian network that is a HC tree. This tree was then used for classifying whether or not the observed marker distribution (either ALS patients or HC) is predicted. To this end, the algorithm computes for each cell the probability q outlined above. We count for each patient the relative number of cells where this probability q is equal to zero (NoZ). This NoZ reflects the similarity in the distribution of mFC markers between a randomly chosen mFC file (either ALS or HC) and the HC data tree. Here, a low NoZ indicates high similarity between an individual and the pooled HC cohort.

**Figure 2:**
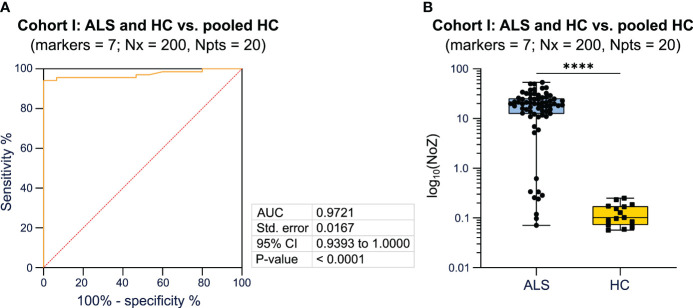
Bayesian analysis differentiates diseased patients from healthy controls. **(A)** ROC analysis including the NoZ for every patient from the ALS and HC cohort. The NoZ was calculated by Bayesian analysis and reflects the similarity in the distribution of mFC markers on a per cell level between an individual and the pooled HC cohort. **(B)** Box plots illustrating the NoZ of the ALS and HC cohort: the box extends from the 25^th^ to 75^th^ percentiles and the median is depicted by the black line in the middle of the box. Min and max values are shown by whiskers. *ALS, amyotrophic lateral sclerosis; AUC, area under the curve; CI, confidence interval; HC, healthy control; NoZ, number of zeros; Npts, number of patients; Nx, bins; ROC, receiver operator curve; Std, standard.* ****p < 0.0001.

In order to assess the performance of the algorithm determined by different parameters (number of included markers, bins, and number of patients) we performed ROC analysis. We observed that the algorithm is able to determine whether a tested individual belongs to the ALS or HC cohort with a high AUC (> 0.90) irrespective of the number of included markers, bins, and number of patients in the pooled HC cohort (**Supplementary Table 1**). To identify the best combination of parameters, we compared AUC and p-values for the different combinations and identified the combination of 7 markers, 200 bins, and 20 patients to demonstrate the highest performance, measured by the AUC, on a significance level of p < 0.0001 (**Supplementary Table 1**). Next, we randomly selected different combinations of 7 markers, which were included in the algorithm. The combination of SSC-A, CD38, CD45RO, CD20, CD11b, CD4, and FSC-A reached the highest AUC (0.97) on a significance level of *p* < 0.0001 (**Figure 2A**; **Supplementary Table 2**). Here, ALS showed a median of 19.661 (0.071-53.526) NoZ while HCs featured a median of 0.102 (0.057-0.250) NoZ (**Figure 2B**). For 4 ALS patients, the NoZ was ≤ 0.25. In turn, 64/68 ALS patients were correctly identified (**Figure 2B**). Furthermore, we counted the relative number of occurrences of cell markers that lead yields Q equal to zero. In this regard, CD4 and CD38 obtained the highest counts (**Supplementary Table 3**). Finally, we explored whether a reduction in the input cell number impacts the performance of the algorithm. We found that a lower input cell number leads to lower AUC values (1 000 cells: AUC = 0.68, p = 0.0346; 10 000 cells: AUC = 0.93, p< 0.0001; 100 000 cells: AUC = 0.93, p < 0.0001; 1 000000 cells: AUC = 0.95, p < 0.0001).

In summary, applying a Bayesian network, we were able to differentiate ALS patients from HC with a high AUC when using mFC raw data as input for the algorithm. The combination of 7 markers, 200 bins, and 20 patients showed the highest AUC on a significance level of p < 0.0001. The markers CD4 and CD38 led most often to the zero probability (Q equal to zero).

### 3.3 Bayesian analysis can reliably distinguish ALS patients and HC using different mFC panels

**Figure 3:**
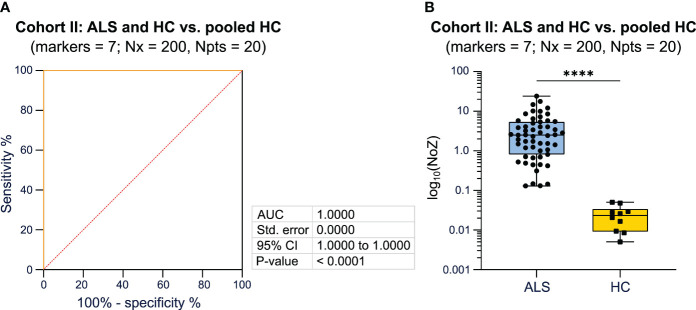
Validation of the utility of Bayesian analysis to classify patients based on the disease status using PB mFC data. **(A)** ROC analysis including the NoZ for every patient from the ALS and HC cohort. The NoZ was calculated by Bayesian analysis and reflects the similarity in the distribution of mFC markers on a per cell level between an individual and the pooled HC cohort. **(B)** Box plots illustrating the NoZ of the ALS and HC cohort: the box extends from the 25^th^ to 75^th^ percentiles and the median is depicted by the black line in the middle of the box. Min and max values are shown by whiskers. *ALS, amyotrophic lateral sclerosis; AUC, area under the curve; CI, confidence interval; HC, healthy control; mFC, multidimensional flow cytometry; NoZ, number of zeros; Npts, number of patients; Nx, bins; PB, peripheral blood; ROC, receiver operator curve; Std, standard.*. ****p < 0.0001.

To validate our approach, we included an independent cohort consisting of 55 ALS patients and 30 HC (Magdeburg cohort). MFC markers assessed in this cohort differed from the Dresden cohort as described in detail in the method section. We again compared the AUC and p-values, calculated by ROC analysis, for different combinations of 7 markers (**Supplementary Table 4**). The combination of the following markers reached the highest AUC (1.0) when comparing ALS patients and HC to the HC tree, respectively: CD14, FSC-A, CCR2, CD16, Lineage, HLA-DR, CXCR1 (**Figure 3A**; **Supplementary Table 4**). The median NoZ for the ALS cohort was 2.519 (0.130 - 24.055) while HC subjects showed a median NoZ of 0.023 (0.005 - 0.050). Correspondingly, the algorithm identified all ALS and HC correctly (**Figure 3B**). We again counted the relative number of occurrences of cell markers that lead yields Q equal to zero. For the Magdeburg cohort, SSC-A and the marker CD20 yielded the highest counts (**Supplementary Table 3**).

Taken together, we were able to validate that Bayesian analysis can reliably differentiate ALS patients from HC when using mFC raw data as input for the algorithm.

### 3.4 Bayesian analysis shows superior ability to Citrus to classify samples based on disease status when including raw, ungated mFC data

**Figure 4:**
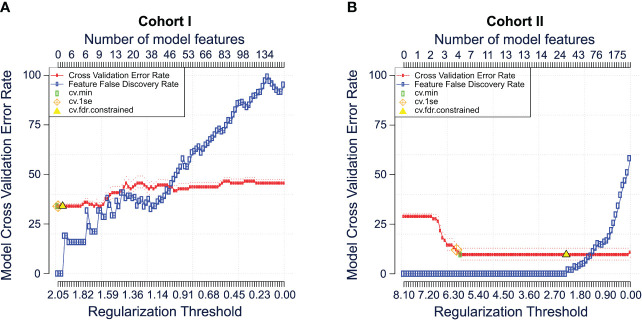
Comparison of the Bayesian analysis with citrus. Cross validation error plots for cohort I **(A)** and cohort II **(B)** illustrating the estimated model accuracy and feature false discovery rate as a function of the model regularization threshold. Plots were generated using the citrus workflow of the OMIQ software from Dotmatics (www.omiq.ai, www.dotmatics.com). The method is described in detail by Bruggner et al. (2). In brief, the nearest shrunken centroid and lasso-regularized logistic regression methods are used to construct classification models. Both methods automatically select subsets of informative features and construct classification models. The number of model regressors is restricted by applying a regularization penalty (λ) for every single feature included in the model. Multiple models are built using different regularization thresholds as it is unknown which subset of cluster features is optimal to stratify the user-specified sample group. Subsequently, cross-validation and permutation tests are performed to calculate and plot the classification error rates and feature false discovery rates of each model. *CV, cross validation; FDR, false discovery rate; Se, standard error*.

Finally, we compared the performance of the Bayesian analysis with a previously validated approach to classify mFC data. For this, the commercially available algorithm ‘Citrus’ was used (see methods). First, mFC data from cohort I (Dresden cohort) were used as workflow input. Citrus constructs several models with increasing complexity by using a range of regularization thresholds. Using *K*-fold cross-validation, error rates of these models are estimated. Cross-validation is performed and a plot showing the fit of all models as a function of the regularization threshold is generated. For the Dresden cohort, the error rate ranged from > 25 to < 50, depending on the regularization threshold (**Figure 4A**). We repeated the analysis for the second cohort (Magdeburg cohort). In this case, the model error rate was lower (< 25 for most regularization thresholds) compared to the Dresden cohort (**Figure 4B**).

In summary, Bayesian analysis shows superior ability to classify mFC data based on the disease status compared to Citrus when including raw, ungated mFC data.

## 4 Discussion

MFC is a valuable tool for analyzing the physical and chemical properties of cells (14–16). However, the analysis of flow cytometry data can be a challenging task, especially when dealing with large datasets. Automated mFC analysis is becoming increasingly important to improve the accuracy, efficiency, and reproducibility of flow cytometry experiments. One of the main advantages of mFC analysis using automated algorithms is the ability to standardize the analysis process and reduce the inter-operator variability. This can lead to more reproducible results and facilitate the comparison of data across different studies. Additionally, automated analysis is time-efficient and resource saving, allowing researchers to focus on more complex analyses and interpretations.

Currently, a plethora of tools are available for automated analysis of mFC data (1, 17). Many of these algorithms employ dimensionality reduction techniques to reduce dataset complexity. While this approach can improve interpretability, dimensionality reduction also introduces a set of limitations: First, these algorithms can result in the loss of information contained in the original high-dimensional data. This can occur because these algorithms collapse multiple dimensions into a smaller set of dimensions, thereby reducing the amount of information available for downstream analysis (18). Second, dimensionality reduction algorithms can be prone to overfitting, particularly when the number of dimensions in the original data is large. This can lead to poor generalization performance when the reduced-dimensional data are used for downstream analysis.

To explore strategies for automated mFC analysis while retaining information on the entire dataset, we constructed an algorithm centered on Bayesian network analysis (19). A potential advantage of Bayesian network analysis is that this algorithm allows to incorporate new patient data into an existing model. As such, a new set of data may be classified by an established Bayesian network without the loss of information due to reduction techniques. Concurrently, Bayesian network analysis is scalable and not hindered by large numbers of cells to be analyzed. Applying this approach, we were able to successfully discriminate ALS patients from HC with a high AUC using mFC raw data as input. The utility of Bayesian analysis in this context could be validated in a second, independent cohort of ALS patients and HC using a different mFC panel. This indicates robust changes in peripheral immune cell profiles of ALS patients compared to HC. In addition, Bayesian network analysis can be used to assess the relative number of occurrences of cell markers that lead yields **Q** equal to zero potentially providing novel biological information about the disease. The lower performance of the algorithm with decreasing input cell numbers could indicate a relevant impact of low abundant cell subsets when differentiating between ALS und HC.

Currently, our algorithm is designed to facilitate a classification task (diseased vs. non-diseased patients). Once established, the Bayesian tree can be used to predict the disease status of new patients. The performance of the algorithm when discriminating clinically relevant differential diagnoses has to be evaluated in future studies to assess the diagnostic benefit of Bayesian network analysis as a complement to the current clinical routine workup. In this context, it has to be acknowledged that different diseases might have similar effects on the peripheral immune response. Thus, choosing the optimal mFC marker combination can be challenging and might be crucial for the performance of the algorithm. In this regard, the combination of cell surface and intracellular mFC markers might prove useful.

Another challenge might be the implementation of Bayesian analysis into the clinical routine workup. Different variables, which can potentially influence the peripheral immune cell profile of patients (e.g., age, sex, comorbidities, medications), as well as technical differences between centers should be taken into consideration. Therefore, multiple center-specific control cohorts considering potential confounding factors might be necessary. Apart from classifying patients based on the disease status, Bayesian analysis has the potential to predict treatment responses or clinical outcomes. Well-characterized, center-specific reference cohorts could be used to establish Bayesian networks and to define the NoZ serving as ‘cut-off’ values between two opposing outcomes (e.g., response to treatment vs. no treatment response or good clinical outcome vs. unfavorable clinical outcome). Subsequently, a new set of data can be classified by this Bayesian network. Thus, applying Bayesian analysis to flow cytometry data opens up manifold novel possibilities which might stimulate future research.

A limitation to the current design is that the immunological parameters driving this classification are difficult to discern. Larger cohorts using similar marker combinations will be necessary to obtain relevant biological information. Bayesian analysis might be beneficial to identify stratifying markers between clinical differential diagnosis, which could improve the pathophysiological understanding of the diseases. Another limitation of this approach might be that only two outcomes can be differentiated by one Bayesian tree. However, to date, reliable diagnostic and prognostic biomarkers are lacking for many diseases. Bayesian analysis could support outcome prediction in the future using PB, which can be obtained easily and non-invasively. Additional studies will be necessary to assess the value of Bayesian network analysis in this context.

## 5 Conclusion

As the field of mFC continues to evolve, it is likely that more sophisticated algorithms and analytical tools will become available for research. These algorithms might benefit from incorporating Bayesian networks as they allow for the inclusion of new patient data without the need for dimensionality reduction. Further scientific effort is needed to standardize automated algorithms used for interpretation of mFC data.

## Data availability statement

The raw data supporting the conclusions of this article will be made available by the authors, without undue reservation.

## Ethics statement

The studies involving human participants were reviewed and approved by Ethikkommission an der Technischen Universität Dresden (EK393122012) and Ethik-Kommission der Otto-von-Guericke-Universität in Magdeburg (07/17 and 11/21). The patients/participants provided their written informed consent to participate in this study.

## Author contributions

SR: Conceptualization, Methodology, Formal analysis, Validation, Writing - Original Draft, Writing - Review and Editing, Visualization; CN: Conceptualization, Methodology, Formal analysis, Validation, Writing - Original Draft, Writing - Review and Editing, Visualization; CS: Writing - Review and Editing, Visualization, SB: Methodology, Writing - Review and Editing; MP: Resources, Writing - Review and Editing; JI: Writing - Review and Editing; KA: Investigation, Writing - Review and Editing; RG: Investigation; AG: Investigation, Writing - Review and Editing; MM: Investigation, Writing - Review and Editing; ID: Resources, Conceptualization, Writing - Review and Editing; SS: Resources, Conceptualization, Writing - Review and Editing; SV: Resources; TZ: Resources, Conceptualization, Writing - Review and Editing; NM: Writing - Review and Editing, Supervision; TR: Writing - Review and Editing, Supervision; SM: Conceptualization, Resources, Writing - Review and Editing, Supervision, Project administration; MH: Conceptualization, Methodology, Software, Formal analysis, Validation, Resources, Writing - Original Draft, Writing - Review and Editing. All authors contributed to the article and approved the submitted version.
