# Supplementary material for: Classifying flow cytometry data using Bayesian analysis helps to distinguish ALS patients from healthy controls
Source: Front Immunol. 2023 Aug 1;14:1198860. doi: 10.3389/fimmu.2023.1198860 (PMC10434536; doi:10.3389/fimmu.2023.1198860)
Supplement: Supplementary file 1 [file DataSheet_1.pdf]

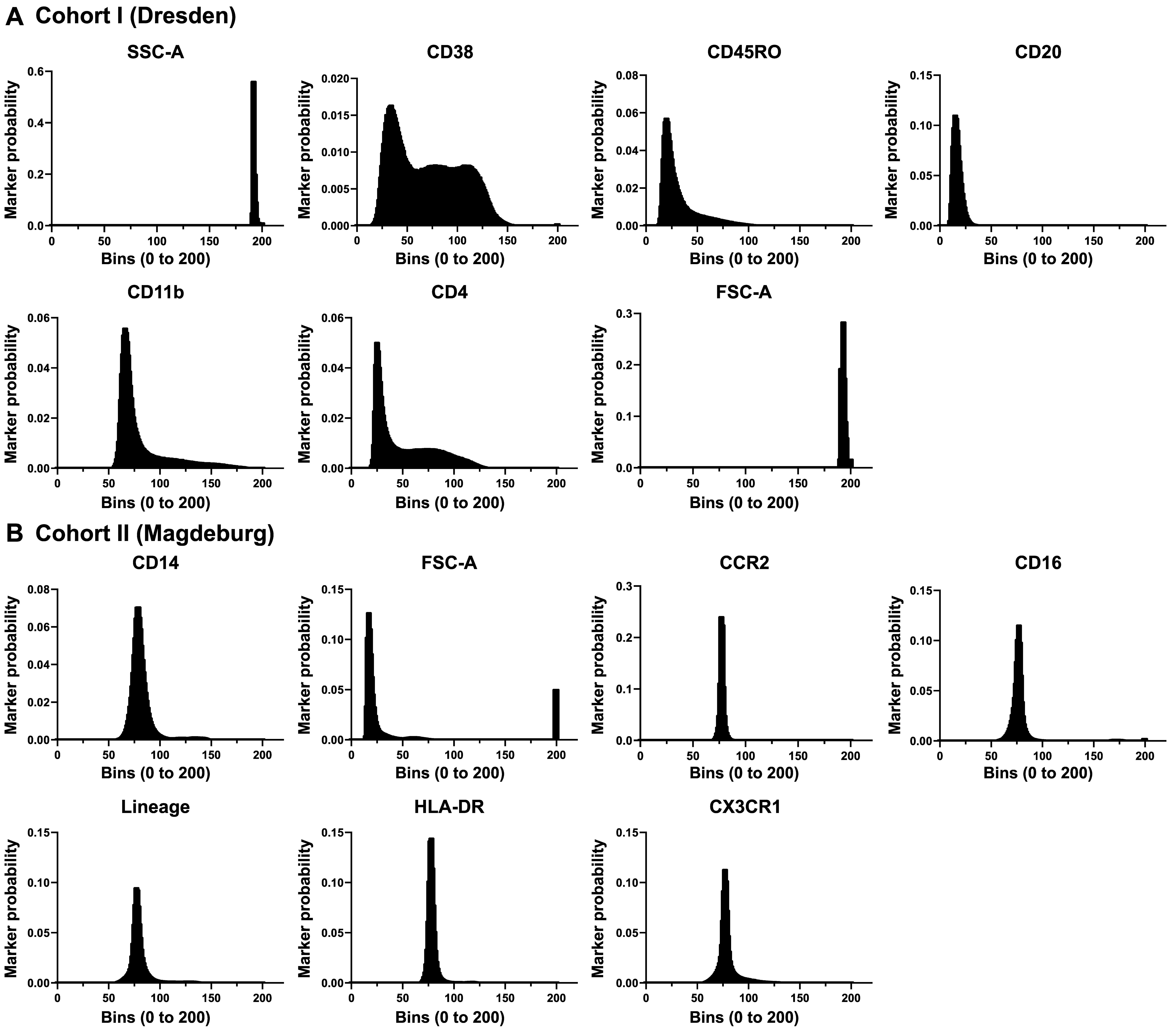

Supplementary Figure 1: Marker probabilities

FSC - forward scatter; SSC - side scatter

Marker	Nx	Npts	AUC	p-value
6	50	30	0.9691	0.0005
**7**	**200**	**20**	**0.9627**	**<0.0001**
6	200	30	0.9603	0.0006
8	50	30	0.9603	0.0006
8	100	30	0.9588	0.0007
6	200	10	0.9576	<0.0001
8	200	30	0.9559	0.0007
8	50	20	0.9544	<0.0001
7	200	10	0.9535	<0.0001
6	100	30	0.9529	0.0008
9	100	30	0.9529	0.0008
9	200	10	0.9529	<0.0001
9	200	30	0.9529	0.0008
8	100	10	0.9524	<0.0001
9	100	20	0.9520	<0.0001
5	200	30	0.9515	0.0008
9	200	20	0.9510	<0.0001
6	100	10	0.9500	<0.0001
9	50	30	0.9500	0.0008
6	50	10	0.9497	<0.0001
9	100	10	0.9474	<0.0001
5	200	20	0.9471	<0.0001
5	100	10	0.9468	<0.0001
7	100	10	0.9459	<0.0001
8	200	10	0.9447	<0.0001
7	100	20	0.9436	<0.0001
6	50	20	0.9431	<0.0001
5	200	10	0.9426	<0.0001
8	50	10	0.9415	<0.0001
5	50	10	0.9409	<0.0001
8	200	20	0.9407	<0.0001
6	100	20	0.9402	<0.0001
5	100	30	0.9397	0.0011
8	100	20	0.9397	<0.0001
6	200	20	0.9382	<0.0001
7	50	30	0.9382	0.0011
9	50	10	0.9371	<0.0001
7	100	30	0.9353	0.0012
5	100	20	0.9309	<0.0001
7	50	20	0.9309	<0.0001
7	50	10	0.9285	<0.0001
5	50	20	0.9245	<0.0001
9	50	20	0.9235	<0.0001
7	200	30	0.9191	0.0019
5	50	30	0.9162	0.002
12	50	30	0.9087	<0.0001
10	50	30	0.9079	<0.0001
11	50	30	0.9063	<0.0001
12	100	30	0.9026	<0.0001
11	200	30	0.9006	<0.0001
10	200	30	0.9002	<0.0001
11	100	30	0.8986	<0.0001
10	100	30	0.8974	<0.0001
12	200	30	0.8909	<0.0001

Supplementary table 1 - Tuning of parameters

ROC analysis including the number of zeros when comparing ALS patients and HC to the HC tree with different numbers of markers, bins, and numbers of patients included in the algorithm. AUC values and p-values were calculated and sorted in a descending order. The best marker, NX, and Npts combination - based on the AUC and p-value - is highlighted.

ALS - amyotrophic lateral sclerosis; AUC - area under the curve; HC - healthy control; Npts - number of patients; Nx - bins; ROC - receiver operator curve

Marker combination	AUC	p-value
**1 = SSC-A**<br>**2 = Alexa Fluor 700-CD38-A**<br>**3 = BV605-CD45RO-A**<br>**4 = FITC-CD20-A**<br>**5 = V450-CD11b-A**<br>**6 = PE-Cy7-CD4-A**<br>**7 = FSC-A**	**0.9721**	**<0.0001**
1 = V450-CD11b-A<br>2 = APC-Cy7-CD3-A<br>3 = PerCP-Cy5-5-CD8-A<br>4 = BV605-CD45RO-A<br>5 = PE-Cy7-CD4-A<br>6 = FSC-A<br>7 = PE-Cy5-5-CD11c-A	0.9652	<0.0001
1 = FITC-CD20-A<br>2 = FSC-A<br>3 = Alexa Fluor 700-CD38-A<br>4 = PE-Cy7-CD4-A<br>5 = PE-CRTH2-A<br>6 = PE-Cy5-5-CD11c-A<br>7 = V450-CD11b-A	0.9647	<0.0001
1 = PE-Cy5-5-CD11c-A<br>2 = APC-Cy7-CD3-A<br>3 = PE-CF594-CCR7-A<br>4 = V500-CD45RA-A<br>5 = SSC-A<br>6 = PerCP-Cy5-5-CD8-A<br>7 = Alexa Fluor 700-CD38-A	0.9642	<0.0001
1 = V500-CD45RA-A<br>2 = SSC-A<br>3 = PE-Cy5-5-CD11c-A<br>4 = V450-CD11b-A<br>5 = PE-CF594-CCR7-A<br>6 = PerCP-Cy5-5-CD8-A<br>7 = FSC-A	0.9618	<0.0001
1 = BV605-CD45RO-A<br>2 = V450-CD11b-A<br>3 = PerCP-Cy5-5-CD8-A<br>4 = PE-Cy7-CD4-A<br>5 = PE-Cy5-5-CD11c-A<br>6 = FSC-A<br>7 = FITC-CD20-A	0.9608	<0.0001
1 = PE-CF594-CCR7-A<br>2 = PE-Cy5-5-CD11c-A<br>3 = APC-Cy7-CD3-A<br>4 = V450-ACD11b-A<br>5 = FSC-A<br>6 = PerCP-Cy5-5-CD8-A<br>7 = Alexa Fluor 700-CD38-A	0.9578	<0.0001

1 = APC-Cy7-CD3-A	0.9564	<0.0001
2 = FSC-A		
3 = PE-Cy7-CD4-A		
4 = V500-CD45RA-A		
5 = SSC-A		
6 = PE-Cy5-5-CD11c-A		
7 = PE-CF594-CCR7-A		
1 = PE-CRTH2-A	0.9559	<0.0001
2 = APC-CD25-A		
3 = PerCP-Cy5-5-CD8-A		
4 = APC-Cy7-CD3-A		
5 = BV605-CD45RO-A		
6 = SSC-A		
7 = V500-CD45RA-A		
1 = PE-Cy5-5-CD11c-A	0.9549	<0.0001
2 = SSC-A		
3 = PE-CF594-CCR7-A		
4 = Alexa Fluor 700-CD38-A		
5 = PE-Cy7-CD4-A		
6 = FSC-A		
7 = V450-CD11b-A		
1 = V500-CD45RA-A	0.9539	<0.0001
2 = SSC-A		
3 = APC-Cy7-CD3-A		
4 = PE-CRTH2-A		
5 = PE-CF594-CCR7-A		
6 = V450-CD11b-A		
7 = PE-Cy7-CD4-A		
1 = PE-CF594-CCR7-A	0.9534	<0.0001
2 = PE-Cy5-5-CD11c-A		
3 = Alexa Fluor 700-CD38-A		
4 = SSC-A		
5 = FSC-A		
6 = FITC-CD20-A		
7 = PerCP-Cy5-5-CD8-A		
1 = V500-CD45RA-A	0.9529	<0.0001
2 = FITC-CD20-A		
3 = FSC-A		
4 = V450-CD11b-A		
5 = PerCP-Cy5-5-CD8-A		
6 = Alexa Fluor 700-CD38-A		
7 = PE-Cy7-CD4-A		
1 = PE-CF594-CCR7-A	0.952	<0.0001
2 = V500-CD45RA-A		
3 = PerCP-Cy5-5-CD8-A		
4 = FSC-A		
5 = FITC-CD20-A		
6 = APC-CD25-A		
7 = PE-CRTH2-A		

1 = PE-CRTH2-A	0.952	<0.0001
2 = FITC-CD20-A		
3 = BV605-CD45RO-A		
4 = PE-Cy7-CD4-A		
5 = PE-CF594-CCR7-A		
6 = FSC-A		
7 = V450-CD11b-A		
1 = PE-CF594-CCR7-A	0.951	<0.0001
2 = BV605-CD45RO-A		
3 = V450-CD11b-A		
4 = PE-Cy5-5-CD11c-A		
5 = PE-Cy7-CD4-A		
6 = FSC-A		
7 = APC-CD25-A		
1 = V500-CD45RA-A	0.951	<0.0001
2 = PE-CF594-CCR7-A		
3 = APC-Cy7-CD3-A		
4 = Alexa Fluor 700-CD38-A		
5 = FITC-CD20-A		
6 = V450-CD11b-A		
7 = PE-Cy7-CD4-A		
1 = PE-CF594-CCR7-A	0.95	<0.0001
2 = PerCP-Cy5-5-CD8-A		
3 = SSC-A		
4 = Alexa Fluor 700-CD38-A		
5 = FSC-A		
6 = V500-CD45RA-A		
7 = PE-Cy7-CD4-A		
1 = APC-CD25-A	0.95	<0.0001
2 = SSC-A		
3 = V500-CD45RA-A		
4 = PE-Cy7-CD4-A		
5 = PE-CRTH2-A		
6 = FITC-CD20-A		
7 = PE-CF594-CCR7-A		
1 = APC-CD25-A	0.95	<0.0001
2 = BV605-CD45RO-A		
3 = PE-Cy5-5-CD11c-A		
4 = APC-Cy7-CD3-A		
5 = PE-CRTH2-A		
6 = PerCP-Cy5-5-CD8-A		
7 = V450-CD11b-A		
1 = PE-Cy7-CD4-A	0.949	<0.0001
2 = BV605-CD45RO-A		
3 = PE-CRTH2-A		
4 = PerCP-Cy5-5-CD8-A		
5 = Alexa Fluor 700-CD38-A		
6 = FSC-A		
7 = V450-CD11b-A		

1 = PE-CRTH2-A<br>2 = BV605-CD45RO-A<br>3 = V500-CD45RA-A<br>4 = PE-CF594-CCR7-A<br>5 = V450-CD11b-A<br>6 = FITC-CD20-A<br>7 = PE-Cy7-CD4-A	0.949	<0.0001
1 = PE-Cy7-CD4-A<br>2 = FITC-CD20-A<br>3 = APC-CD25-A<br>4 = PerCP-Cy5-5-CD8-A<br>5 = FSC-A<br>6 = PE-Cy5-5-CD11c-A<br>7 = Alexa Fluor 700-CD38-A	0.948	<0.0001
1 = BV605-CD45RO-A<br>2 = Alexa Fluor 700-CD38-A<br>3 = PE-Cy7-CD4-A<br>4 = SSC-A<br>5 = PE-CF594-CCR7-A<br>6 = PE-Cy5-5-CD11c-A<br>7 = V500-CD45RA-A	0.948	<0.0001
1 = V500-CD45RA-A<br>2 = SSC-A<br>3 = BV605-CD45RO-A<br>4 = APC-CD25-A<br>5 = FSC-A<br>6 = FITC-CD20-A<br>7 = Alexa Fluor 700-CD38-A	0.9461	<0.0001
1 = PE-Cy7-CD4-A<br>2 = FSC-A<br>3 = FITC-CD20-A<br>4 = V500-CD45RA-A<br>5 = PE-CF594-CCR7-A<br>6 = APC-CD25-A<br>7 = Alexa Fluor 700-CD38-A	0.9451	<0.0001
1 = FSC-A<br>2 = SSC-A<br>3 = V450-CD11b-A<br>4 = V500-CD45RA-A<br>5 = BV605-CD45RO-A<br>6 = APC-CD25-A<br>7 = Alexa Fluor 700-CD38-A	0.9441	<0.0001
1 = PE-CRTH2-A<br>2 = FSC-A<br>3 = PE-CF594-CCR7-A<br>4 = V450-CD11b-A<br>5 = FITC-CD20-A<br>6 = PE-Cy5-5-CD11c-A<br>7 = PerCP-Cy5-5-CD8-A	0.9441	<0.0001

1 = V450-CD11b-A<br>2 = PE-Cy5-5-CD11c-A<br>3 = PE-Cy7-CD4-A<br>4 = FITC-CD20-A<br>5 = SSC-A<br>6 = APC-Cy7-CD3-A<br>7 = Alexa Fluor 700-CD38-A	0.9436	<0.0001
1 = APC-CD25-A<br>2 = Alexa Fluor 700-CD38-A<br>3 = V450-CD11b-A<br>4 = FITC-CD20-A<br>5 = APC-Cy7-CD3-A<br>6 = PerCP-Cy5-5-CD8-A<br>7 = BV605-CD45RO-A	0.9431	<0.0001
1 = V450-CD11b-A<br>2 = PE-Cy5-5-CD11c-A<br>3 = PE-CF594-CCR7-A<br>4 = Alexa Fluor 700-CD38-A<br>5 = PE-Cy7-CD4-A<br>6 = APC-Cy7-CD3-A<br>7 = APC-CD25-A	0.9382	<0.0001

Supplementary table 2 - Tuning of marker combination
ROC analysis including the number of zeros when comparing ALS patients and HC to the HC tree with different combinations of 7 markers included in the algorithm. AUC values and p-values were calculated and sorted in a descending order. The best marker combination - based on the AUC and p-value - is highlighted.
ALS - amyotrophic lateral sclerosis; AUC - area under the curve; HC - healthy control; ROC - receiver operator curve

Cohort	Marker	Rel. no occurrences of cell markers that yield Q equal to zero
I (Dresden)	Marker 6: PE-Cy7-CD4-A	1064493
	Marker 2: Alexa Fluor 700-CD38-A	975188
	Marker 3: BV605-CD45RO-A	232353
	Marker 5: V450-CD11b-A	182740
	Marker 4: FITC-CD20-A	39977
	Marker 7: FSC-A	13795
	Marker 1: SSC-A	13510
II (Magdeburg)	Marker 1: SSC-A	493507
	Marker 4: FITC-CD20-A	465733
	Marker 6: PE-Cy7-CD4-A	215814
	Marker 5: V450-CD11b-A	123914
	Marker 3: BV605-CD45RO-A	81804
	Marker 7: FSC-A	11671
	Marker 2: Alexa Fluor 700-CD38-A	6007

Supplementary table 3 - Statistics on the markers that yield a zero probability
FSC - forward scatter; no - number of; SSC - side scatter

Marker combination	AUC	p-value
**1 = CD14-BV510-A**	**1.0000**	**<0.0001**
**2 = FSC-A**		
**3 = CCR2-R-PE-A**		
**4 = CD16-FITC-A**		
**5 = Lineage-Alexa Fluor* 700-A**		
**6 = HLA-DR-PerCP-Cy5.5-A**		
**7 = CX3CR1-PE-Cy7-A**		
1 = CD86-APC-A	0.9982	<0.0001
2 = CD16-FITC-A		
3 = SSC-A		
4 = DUMP-Alexa Fluor* 700-A		
5 = CD14-BV510-A		
6 = FSC-A		
7 = HLA-DR-PerCP-Cy5.5-A		
1 = CD16-FITC-A	0.9982	<0.0001
2 = SSC-A		
3 = HLA-DR-PerCP-Cy5.5-A		
4 = CX3CR1-PE-Cy7-A		
5 = FSC-A		
6 = CD86-APC-A		
7 = CCR2-R-PE-A		
1 = FSC-A	0.9821	<0.0001
2 = SSC-A		
3 = CD16-FITC-A		
4 = HLA-DR-PerCP-Cy5.5-A		
5 = CX3CR1-PE-Cy7-A		
6 = CCR2-R-PE-A		
7 = CD86-APC-A		
1 = SSC-A	0.9804	<0.0001
2 = Lineage-Alexa Fluor* 700-A		
3 = CD86-APC-A		
4 = CX3CR1-PE-Cy7-A		
5 = CD14-BV510-A		
6 = CCR2-R-PE-A		
7 = HLA-DR-PerCP-Cy5.5-A		
1 = CX3CR1-PE-Cy7-A	0.9768	<0.0001
2 = FSC-A		
3 = HLA-DR-PerCP-Cy5.5-A		
4 = CD14-BV510-A		
5 = CD86-APC-A		
6 = Lineage-Alexa Fluor* 700-A		
7 = SSC-A		
1 = CD16-FITC-A	0.9643	<0.0001
2 = CCR2-R-PE-A		
3 = FSC-A		
4 = SSC-A		
5 = CD86-APC-A		
6 = CX3CR1-PE-Cy7-A		
7 = CD14-BV510-A		

1 = CD86-APC-A<br>2 = FSC-A<br>3 = Lineage-Alexa Fluor* 700-A<br>4 = SSC-A<br>5 = CX3CR1-PE-Cy7-A<br>6 = CD14-BV510-A<br>7 = CCR2-R-PE-A	0.9357	<0.0001
1 = CD16-FITC-A<br>2 = Lineage-Alexa Fluor* 700-A<br>3 = CX3CR1-PE-Cy7-A<br>4 = HLA-DR-PerCP-Cy5.5-A<br>5 = CCR2-R-PE-A<br>6 = SSC-A<br>7 = CD14-BV510-A	0.9321	<0.0001
1 = SSC-A<br>2 = Lineage-Alexa Fluor* 700-A<br>3 = CD14-BV510-A<br>4 = CCR2-R-PE-A<br>5 = FSC-A<br>6 = CX3CR1-PE-Cy7-A<br>7 = HLA-DR-PerCP-Cy5.5-A	0.8357	0.0008

Supplementary table 4 - Tuning of marker combination (cohort 2)
ROC analysis including the number of zeros when comparing ALS patients and HC to the HC tree with different combinations of 7 markers included in the algorithm. AUC values and p-values were calculated and sorted in a descending order. The best marker combination - based on the AUC and p-value - is highlighted.
ALS - amyotrophic lateral sclerosis; AUC - area under the curve; HC - healthy control; ROC - receiver operator curve
